# Hair-lip and Cleft Palate

**Published:** 1863-11

**Authors:** 


Hair-lip and Cleft Palate.—At a late sitting of the Academy of Sciences, Dr. Sédillot read a paper on a remarkable case of hare-lip, complicated with a wide split existing in the roof of the mouth, or hard palate. Dr. Sedillot, following Prof. Langenbeck's method, first detached the periosteum from the two osseous surfaces of the palate right and left, and joined them together, so as to close the hiatus. This was done on the 23d of May: on the 30th the roof of the mouth was restored, except a small fissure still remaining at the further end. As it would have been the hight of imprudence to renew the operation before the newly-closed parts were perfectly consolidated, the operator waited until the 26th of August, when the fissure was closed by means of a portion of periosteum borrowed from the adjacent part. It was remarked that the portion previously restored had undergone no ossification, but had nevertheless been covered by a new mucous membrane, while the space which had been denuded of its periosteum had received a fresh coating.

